# Bentley's Miscellany and Mad Doctors

**Published:** 1850-01-01

**Authors:** 


### BENTLEY'S MISCELLANY AND MAD DOCTORS.

THERE is a popular charivari—a hue and cry raised abroad against "Mad Doctors;" they are pointed at and hunted down, like the unfortunate mad dog which Goldsmith described in his celebrated Epitaph. Whence does this arise? Is it from ignorance, or from that love of the ridiculous which is one of the characteristic features of the present age? True it is that prejudices founded originally upon matters of fact are always difficult to eradicate. The persistency of mental impressions would appear to be transmitted from one generation to another; and hence some uneducated people may imagine that the poor lunatic is still immured in a prison-like cell and chained from the wall to his bed of straw, and they may, to complete the picture of his misery, also suppose him to be tyrannized over by a house doctor, who has all the heartlessness of a common gaoler and the ruthlessness of a hired turnkey. But the possibility of this—all commonly well-informed persons, particularly literary men, know to be at present utterly false; we therefore marvel that we should find in so highly respectable and excellent a contemporary as "Bentley's Miscellany" an article perpetuating, if not, to a certain degree, originating such misrepresentations. Instead, however, of accusing the author of ignorance, we are rather disposed to attribute his defalcation to that spirit of caricature which now-a-days gives a grotesque aspect to the simplest, as well as to the most serious incidents of daily life. Indeed, we are inclined to think that the extravagantly comic vein which now prevails deteriorates from the interests of science and the true dignity of literature. "Vive la bagatelle" was Dean Swift's frequent exclamation; but let it be in proper place and season;—and we entertain a strong conviction that human suffering, under whatever calamitous form it may be developed, ought to be held sacred from ridicule.

These observations have heen suggested by the article referred to—viz., the 31st Chapter from the "Note Book of a Coroner's Clerk," by the author of "Experiences of a Gaol Chaplain." We may indeed very conscientiously object *in limine* to any series under such a title, for the duties of a coroner, who is called upon to investigate into the causes of sudden death, are, it is obvious, of too distressing a nature to be trifled with even in fiction. We cannot imagine so unfeeling a spectacle as that of a mock inquest introduced into the middle of a broad farce—yet we are here promised a description of one in the next chapter; but the experience of a Gaol Chaplain may somewhat have blunted the finer susceptibilities of the heart. For this reason we need not be surprised to find our author—partly, we presume, from ignorance and partly from a desire of displaying the coruscations of his subtle wit—joining in the outcry raised against "mad doctors;" but upon this point we would fain have a few words with this learned Theban.

To designate a physician by the *pre-nomen* of any class of diseases he may specially treat is a manifest absurdity. We may as well talk of a lung doctor—a stomach doctor—or a kidney doctor, as of a "mad doctor." Or to change the illustration, we may, looking to the legal profession upon the same principle, speak of a petty-larceny attorney—a burglary solicitor—or a forging attorney-general. This would really be as apposite as the the term "mad doctor" thus vulgarly applied to those professional men who devote their industry and talents to the study and treatment of one of the most difficult and important departments of medical science. We do not find in Germany or in France any such disposition to underrate the *status* of such men as Ideler, Jacobi, Foville, Leuret, Mitivié, Parchappe, and others, who toil in the same field; yet in England, as if we were still oppressed by the superstition and darkness of the middle ages the same class of men are denounced as empirics—"mad—nothing else but mad." This is the moral propounded by the coroner's clerk, who next pretends to give an account of the proceedings of a Commission in Lunacy, which are totally unlike anything of the kind we ever witnessed. It is difficult to say, indeed, which of the imaginary characters he describes giving evidence in the case is most absurd. The "mad doctor" is thus portrayed, probably after the type of some Newgate official, which the author's experience or imagination may have suggested :—" He" [Doctor Quoddums] "was a sour looking elderly gentleman, who heightened the forbidding expression of sharp angular features by a constant and ominous frown. Very voluble and very prompt was he in his replies. * * * The doctor's gloomy features relaxed into a lively green when he uttered this dictum—he smacked his lean lips and looked around him with the triumphant air of a man who had said something pre-eminently felicitous and appropriate." * * * "Sir !" says Quoddums, and knitting his brows fiercely and frowning upon Bohun as if he would annihilate him, "I am versed in Insanity and I practice among the mad. * * * I am not to be deceived—it's an impossibility. I found my decision upon data which cannot mislead me. *I judge by the eye—its colour—its sadness—its unrest ; the involuntary movements of the hands tell me much—the action of the muscles of the mouth tell me more.* I want no words—no language—no sounds to influence my decision !" Here Dr. Quoddums swelled out his rude ungainly figure, put a thumb into each pocket of his perfect velvet waistcoat, and faced his auditory, revolving round as it were on a pivot to each point of the compass. "Once adopted, it is immovable, and I pronounce unhesitatingly Sir Philip Grey de Fontenay to be insane."

Our readers will observe that the author here somewhat defeats the objects of his own satire, as the expression of the eye, the involuntary movements and actions of the muscles of the mouth are physiognomical signs for the guidance, with other symptoms, of every pathologist; but why this exaggerated account of the *personnel* of the doctor should be given does not appear, excepting for the purpose of that species of ridicule which is of the lowest order—viz., personal ridicule. The young man who is the supposed lunatic, Sir Philip Grey de Fontenay, is next brought into court, and where is the humanity, we would fain ask, of such a description as the following ?—"He looked pale, helpless, feeble, agitated, could with difficulty articulate, and his reply to the courteous and reassuring terms in which the commissioners addressed him, if reply indeed there were, was a whisper." We all know that patients are unhappily so reduced by great and prolonged mental suffering ; but it is too painful a picture for a gratuitously fictitious representation. The afflictions of mankind ought to be held sacred, and perhaps there is no disease which should be more tenderly treated than the one which is here so unkindly and ruthlessly dealt with. We have next, as before a Commission of Lunacy, a mock examination into the religious creed and conduct of the unfortunate young man, who, to heighten the dramatic effect of the tale, is supposed to be a clergyman, and questions are asked as to the manner in which he performs his clerical duties, and his observance of the anniversary of his ordination. But we must here abruptly break off, for the incidents of such a narration are, to our mind, too melancholy for repetition; indeed, we confess that we have no admiration for that description of wit which neither "points a moral nor adorns a tale." The author, as we have premised, promises in the next chapter an account of the inquest upon the remains of poor Sir Philip Grey de Fontenay; but before he proceeds we should recommend him to reflect that his experience, fictitious or real, as a chaplain in a gaol, was probably among criminals who were in "robust unfeeling health," but the sufferings of those whom it may please Providence to afflict with mental disease assume a very different aspect. The sorrows of the house of mourning—nay, the distress of the individual mind, struggling in the shadow of its own eclipse with sad and misshapen thoughts, ought not to be dilated upon by the pen or pencil of the caricaturist; and we hope the accomplished editor of "Bentley's Miscellany," who is so highly and deservedly esteemed in the literary world, will permit us to assure him that "mad doctors" do not in the least degree resemble the portrait which his contributor has drawn of them, and that all persons who have had any experience in the treatment of the insane feel they have so strong a claim upon our sympathies that we cannot contemplate even the imaginary delineation of their malady without feeling deep regret that so painful a subject should be wantonly travestied into the details of an uninteresting fiction.

---

